# Development and Validation of a New Score to Assess the Risk of Posttransplantation Diabetes Mellitus in Kidney Transplant Recipients

**DOI:** 10.1097/TXD.0000000000001558

**Published:** 2023-11-08

**Authors:** Lina Maria Serna-Higuita, Maria Carolina Isaza-López, Gilma Norela Hernández-Herrera, Angelica Maria Serna-Campuzano, John Fredy Nieto-Rios, Nils Heyne, Martina Guthoff

**Affiliations:** 1 Department of Clinical Epidemiology and Applied Biostatistics, University Hospital Tübingen, Germany.; 2 Faculty of Medicine, University of Antioquia, Medellín, Colombia.; 3 Department of Nephrology, Hospital Pablo Tobón Uribe, Medellín, Colombia.; 4 Department of Diabetology, Endocrinology, Nephrology, University of Tübingen, Tübingen, Germany.; 5 Institute for Diabetes Research and Metabolic Diseases of the Helmholtz Center Munich at the University of Tübingen, Tübingen, Germany.; 6 German Center for Diabetes Research (DZD e.V.), Neuherberg, Germany.

## Abstract

**Background.:**

Posttransplantation diabetes mellitus (PTDM) is a serious complication of solid organ transplantation. It is associated with major adverse cardiovascular events, which are a leading cause of morbidity and mortality in transplant patients. This study aimed to develop and validate a score to predict the risk of PTDM in kidney transplant recipients.

**Methods.:**

A single-center retrospective cohort study was conducted in a tertiary care hospital in Medellín, Colombia, between 2005 and 2019. Data from 727 kidney transplant recipients were used to develop a risk prediction model. Significant predictors with competing risks were identified using time-dependent Cox proportional hazard regression models. To build the prediction model, the score for each variable was weighted using calculated regression coefficients. External validation was performed using independent data, including 198 kidney transplant recipients from Tübingen, Germany.

**Results.:**

Among the 727 kidney transplant recipients, 122 developed PTDM. The predictive model was based on 5 predictors (age, gender, body mass index, tacrolimus therapy, and transient posttransplantation hyperglycemia) and exhibited good predictive performance (C-index: 0.7 [95% confidence interval, 0.65-0.76]). The risk score, which included 33 patients with PTDM, was used as a validation data set. The results showed good discrimination (C-index: 0.72 [95% confidence interval, 0.62-0.84]). The Brier score and calibration plot demonstrated an acceptable fit capability in external validation.

**Conclusions.:**

We proposed and validated a prognostic model to predict the risk of PTDM, which performed well in discrimination and calibration, and is a simple score for use and implementation by means of a nomogram for routine clinical application.

## Graphical abstract

**Figure:**
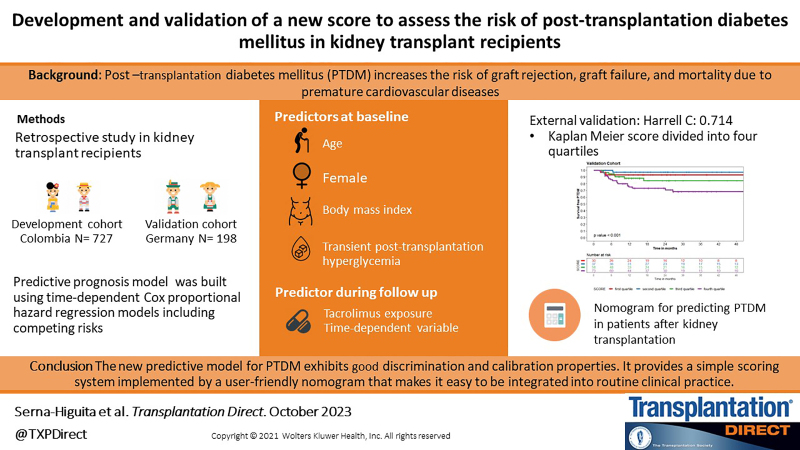

Kidney transplantation is established as the gold-standard therapy of choice for eligible patients with end-stage renal disease with superior patient survival compared with dialysis.^1^ However, it is associated with short- and long-term complications.^2,3^ Posttransplantation diabetes mellitus (PTDM) is a recognized metabolic complication of solid organ transplantation.^4,5^ The prevalence of PTDM varies (10%–46%) depending on many factors, such as the type of transplant and time points evaluated after transplantation.^2,6^ PTDM has been reported to occur in 4% to 25% of renal recipients, 2.5% to 25% of liver transplant recipients, and 4% to 40% of heart transplant recipients.^5,7,8^ The risk of developing PTDM is the highest within the first y after transplantation, with a cumulative incidence of 16%.^3,5,7^

PTDM is a disease distinct from type 1 and type 2 diabetes mellitus. The pathophysiology of PTDM is multifactorial,^2,9,10^ involving not only genetic, environmental, and physiological factors that are related to insulin resistance but also impaired insulin secretion because of β-cell damage.^8,11^ Multiple modifiable and nonmodifiable risk factors have been implicated in the risk of PTDM,^3,6^ such as age, male gender, higher body mass index (BMI), hepatitis C virus infection, hypomagnesemia, genetic susceptibility, reduced physical activity, and unhealthy diet.^3,11^ Immunosuppressive therapy used, such as corticosteroids and calcineurin inhibitors, after transplantation may also increase the risk of PTDM.^11^

The clinical relevance of PTDM lies in its impact on posttransplant outcomes.^8^ Several studies have shown that PTDM increases risk of severe infections and early cardiovascular complications after transplantation.^1,6,11^ PTDM also increases the risk of graft rejection, graft failure, and mortality because of premature cardiovascular diseases.^2,4,7,9,11-13^ A retrospective analysis of the United States Renal Data System, which included >11 000 kidney transplant recipients, showed that PTDM is an independent predictor of mortality and graft failure.^14^ PTDM is also a major cause of chronic kidney disease in liver transplants.^11,15^

To date, no study has shown that treatment of PTDM can reduce the morbidity and mortality in these patients. In fact, the presence of PTDM itself is associated with cardiovascular outcomes independent of treatment.^16^ Therefore, the identification of patients at risk for PTDM might help to guide the implementation of strategies to prevent PTDM,^1,2,8^ including lifestyle modifications, early pharmacological treatment,^1^ and, potentially, the use of less diabetogenic therapy as an immunosuppressive regimen in low immunologic risk patients.^2^ Currently, 2 risk prediction models for PTDM have been developed.^1,17,18^ The first model developed by Chakkera et al included variables of the pretransplant period; however, this score did not consider specific risk factors after transplantation, in particular, transient posttransplantation hyperglycemia and the use of tacrolimus,^17^ which has been identified as a factor that increases the risk of PTDM^1,19^; and the second scale included the use of tacrolimus but only during the first 3 mo after kidney transplantation.^1^ Our study aimed to explore the clinical risk factors related to PTDM, including not only transient posttransplantation hyperglycemia but also tacrolimus use throughout follow-up, to build a predictive risk model for early detection of kidney transplant recipients at high risk for PTDM.

## MATERIALS AND METHODS

### Study Design and Patient Selection

This was a retrospective cohort study of kidney transplant recipients. The derivation cohort was a single-center cohort of patients aged older than 18 y who underwent their first kidney transplantation between January 2005 and December 2019 at Pablo Tobón Uribe Hospital, Medellín, Colombia. We excluded patients with a history of diabetes mellitus before kidney transplantation and those with <1 y of follow-up. Patients were followed up until the onset of PTDM or otherwise censored at a loss to follow-up or at the end of the study. The final follow-up period was December 2020. The external validation cohort included kidney transplant recipients from Tübingen University Hospital, Germany, who underwent kidney transplantation between January 2007 and December 2013 without a history of diabetes mellitus before transplantation. Patients aged younger than 18 y and recipients of combined organ transplantations were excluded.

### Data Collection

Clinical information, including demographic data, comorbidities, and laboratory tests, was collected from the electronic medical record system and from the Renal Transplant Scientific Registry of the Hospital Pablo Tobón Uribe. Multiple sources were used to identify the onset of PTDM, such as laboratory test data (blood biochemical examination) 42 d after transplantation and clinical diagnosis in electronic medical charts, in which data on fasting plasma glucose and glycated hemoglobin (HbA1c) levels were collected during the follow-up as well as clinical outcomes such as graft and patient survival. Data on prescribed medications, including immunosuppressive therapy, were extracted from the electronic database of the hospital from 2005 to 2020, thus allowing immunosuppressive therapy to vary over time at “posttransplantation intervals.”

In the validation cohort of Tübingen, a longitudinal assessment of glucose metabolism (fasting plasma glucose and HbA1c levels) was performed. Additionally, the patient and transplant characteristics (such as immunosuppressive therapy) were assessed.

### Clinical Endpoint (PTDM)

The primary endpoint of this study was to evaluate the independent prognostic factors associated with PTDM. Patients were defined as having new-onset diabetes if they met at least 1 of the following criteria (World Health Organization and American Diabetes Association): (1) fasting blood glucose  ≥ 126 mg/dL, (2) random plasma glucose >200 mg/dL, (3) 2-h plasma glucose ≥200 mg/dL during an oral glucose tolerance test, or (4) HbA1c ≥ 6.5%.^4,6,13,20^ PTDM status was evaluated 42 d after transplantation (derivation cohort). According to the diagnostic criteria for PTDM, recipients who met the inclusion criteria were divided into 2 groups: PTDM and non-PTDM groups. Posttransplant transient hyperglycemia was defined as fasting blood glucose values ≥126 mg/dL or glycosylated hemoglobin >6.5% in the first wks after kidney transplantation.

### Sample Size Estimation

A post hoc power calculation was performed by assuming exponential survival times.^21^ For a fixed sample of 727 patients, an exponential maximum likelihood test of equality of survival curves with a 0.05 two-sided significance level (α) will have 80% power to detect the difference between the exponential parameter λ1 of 0.105 (1-y PTDM event rate equal to 10%) and an exponential parameter λ2 of 0.062 (1-y PTDM event rate 5.5%), and thus a constant hazard ratio (HR) of 1.70. Furthermore, the sample size estimation assumes an average observation time of 2 y.

For the derivation cohort, the sample size had a power of 99%, whereas for the external validation cohort, it was 86%.

### Statistical Analysis

Summary statistics were used to describe the characteristic data of the PTDM and non-PTDM groups. Categorical variables were expressed as absolute and relative frequencies. Continuous variables were described as mean and SD or median and interquartile range according to the distribution of the data, which was assessed using skewness and kurtosis, as well as visual inspection using QQ plots, box plots, and histograms.

The primary endpoint of PTDM was analyzed using cumulative incidence, adjusting for competing risks.^22^ Graft loss and all cause-death were considered competing risk events. The Fine and Gray subdistribution HR (_sd_HR) was used to identify independent factors associated with the risk of PTDM.^23^ A time-dependent Cox regression model was also performed to evaluate the differences in the hazard risk of immunosuppressive therapy on PTDM.^24,25^ Immunosuppressive therapy (cyclosporine, tacrolimus, mycophenolate mofetil, azathioprine, mammalian target of rapamycin inhibitor, such as sirolimus and everolimus) was defined in a time-varying manner, whereby the periods were divided into time intervals after each switching treatment discontinuation.

Prespecified predictors were included on the basis of the published literature related to the onset of PTDM. Variable selection included screening univariate associations (*P* < 0.1) and backward selection using all eligible baseline variables.^26^ Based on the covariates selected, multivariable time-dependent Cox regression models were used to assess HRs and 95% confidence intervals (CIs) for PTDM. To address the missing potential censoring information from the competing risk failure time, we used the model described by Ruan et al,^27^ this method uses a nonparametric multiple imputation method that recovers the missing potential censoring information in the competing risk failure times, so the analysis of time-varying covariates can be applied.^27^ Interactions between the independent covariates were tested using the final model. The linearity of the relationship between the variables and the outcome was evaluated by plotting martingale residuals. The collinearity of the independent variables was evaluated using a correlation matrix. The proportional hazard assumption was tested by plotting the Schoenfeld residuals. The fit of each model was evaluated using the Akaike information criterion and the chi-square goodness of fit statistic.

Discrimination of the prognostic model was evaluated by calculating the concordance Harrell’s C-index (C-index), time-dependent receiver operator characteristics (tROC), and area under these curves (AUC^C/D^),^28^ as well as the concordance probability estimated statistic (CPE, Gonen and Heller’s K-index), which is the best choice in time-to-event data studies.^29^ Calibration was evaluated using Brier scores and calibration plots.^30^ A Brier score of <0.25 indicates that the model can correctly predict the occurrence of the target event, and the closer the score is to 0, the better the model performance.^31^ The bootstrapping sampling method (1000 resamples) was used for internal validation to evaluate the accuracy of the model and reduce overfitting.^32,33^ A prognostic nomogram was constructed on the basis of the multivariate analysis of the validation cohort and was used to predict the 6-, 12-, 18-, and 24-mo risk of PTDM in the presence of competing risks.^34^ Based on the calculated score, the patients were divided into quartiles, and each risk represented a different prognosis. Kaplan-Meier survival analysis was used to generate survival curves, and log-rank tests were used for comparisons between groups.

Multiple-chained equation imputation was used to address the missing values of candidate predictor variables. Fifty imputations were implemented to obtain accurate estimates. Estimates were pooled according to Rubin’s rules.^35,36^

### External Validation

The external validity of the model was assessed using a testing data set. Discrimination was evaluated using the area under the ROC curves, and Harrell’s C-index. Calibration was evaluated using the Brier score and calibration plots. The developed score was then categorized using quartile values in the training cohort as thresholds, and sensitivity, specificity, positive predictive value, and negative predictive value were calculated at 6, 12, 24, 36, and 48 mo.

For all analyses, a 2-tailed *P* value of <0.05 was considered statistically significant. SPSS software (version 28.0; IBM, Chicago, IL) and R (version 4.1) software were used.

### Ethical Considerations

This study was approved by the Ethics Committee of the Pablo Tobón Uribe Hospital (Medellín, Colombia) and the University of Tübingen and was conducted in accordance with the principles of the Declaration of Helsinki. Finally, our article complies with the Transparent Reporting of a multivariable prediction model for Individual Prognosis Or Diagnosis statement.^37^

## RESULTS

**FIGURE 1.**
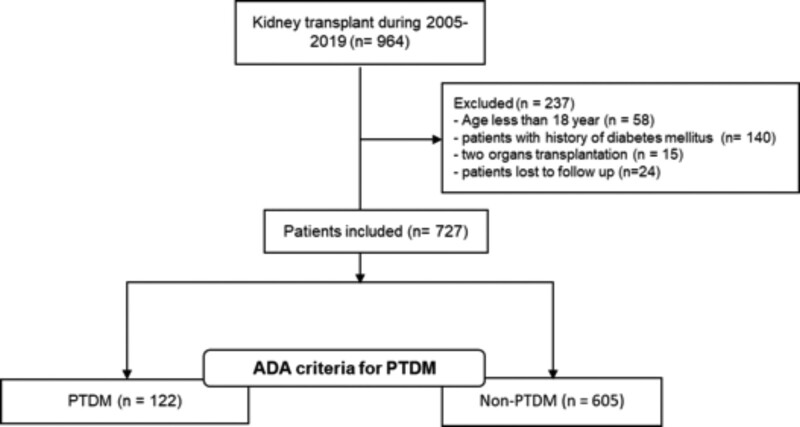
Flowchart cohort. Study flowchart of patient enrollment in the derivation cohort. ADA, American Diabetes Association; PTDM, posttransplantation diabetes.

A total of 964 patients underwent kidney transplantation between 2005 and 2019 in Medellín, Colombia. Among them, 237 were excluded; the reasons for exclusion are shown in Figure 1. Overall, 727 patients were included in the derivation cohort. The cumulative incidence of PTDM at 1, 2, and 4 y after kidney transplantation was 7.9%, 10.5%, and 15.9%, respectively.

Patient characteristics are shown in Table 1. The median age at the time of transplantation was 42 y (range, 33–52 y), and 419 (57.6%) were male. There were 605 and 122 patients in the non-PTDM and PTDM groups, respectively. Patients who developed PTDM were older, with a higher BMI and higher frequency transient posttransplantation hyperglycemia than those in the non-PTDM group. The immunosuppressive regimen and modification therapy are shown graphically for the first 4 y of follow-up (**Figure S1, SDC,** http://links.lww.com/TXD/A592).

**TABLE 1.** Baseline characteristics of the cohort stratified by PTDM (n = 727)

	Total cohort		PTDM			
	(N = 727)		Non-PTDM (n = 605)		PTDM (n = 122)	
Baseline characteristics	Median	IQR	Median	IQR	Median	IQR
Age, y	42	33–52	42	31–51	49	40–58
Weight, kg	74	64–84.6	73.2	63.3–84	77.7	67–88.9
Body mass index, kg/m^2^	**27**	24–29.9	26.6	23.7–29.4	28.5	25.2–32.3
	**n**	**%**	**n**	**%**	**n**	**%**
Cause of CKD						
Autoimmune disorders and glomerulopathies	215	29.6	173	29.1	42	35.3
Ciliopathies	60	8.3	50	8.4	10	8.4
CAKUT	28	3.9	27	4.5	1	0.8
Interstitial nephritis	27	3.7	22	3.7	5	4.2
Nephrolothiasis	10	1.4	4	0.7	6	5
Others	25	3.5	22	3.8	2	1.7
Unknown	348	47.9	296	49.8	52	43.7
	**n**	**%**	**n**	**%**	**n**	**%**
Gender						
Female	308	42.4	249	41.1	59	48.3
Male	419	57.6	356	58.8	63	51.6
	**Median**	**IQR**	**Median**	**IQR**	**Median**	**IQR**
Blood glucose level						
Fasting plasma glucose, mg/dL^*a*^	128	120–151	127	108–147	137	112–166
	**n**	**%**	**n**	**%**	**n**	**%**
Hyperglycemia^*a*^	411	59.3	328	56.9	83	70.9
Insulin therapy^*b*^	76	14.3	54	12	22	27.5
	**Median**	**IQR**	**Median**	**IQR**	**Median**	**IQR**
Blood lipid levels, mg/dL						
Total cholesterol	213	172–251	206	168–245	235	199–269
Low-density lipoprotein	121	94–155	117	93.6–152	147	106–171
High-density lipoprotein	42	35–51	42	35–51	42	34.7–54
Triglycerides	186	129–278	172	125–255	221	160–316
	**n**	**%**	**n**	**%**	**n**	**%**
Dyslipidemia	275	97.1	216	96.8	59	98.3
	**n**	**%**	**n**	**%**	**n**	**%**
Induction therapy						
Thymoglobulin/alemtuzumab	430	75	358	76	72	68.5
Basiliximab/daclizumab	121	21	95	20	26	24
Nontherapy	22	3.8	15	3.2	7	6.6
Follow up, d	767	191–1753	777	188–1785	725	196–1607
	**n**	**%**	**n**	**%**	**n**	**%**
Secondary endpoint (n = 720)						
Acute rejection	135	18.7	111	18.5	24	29.8
Graft lost	100	13.8	74	12.3	26	21.4
Death	30	4.1	21	3.5	9	7.4

^*a*^transient posttransplantation hyperglycemia.

^*b*^insulin therapy during the first wk posttransplantation.

CAKUT, congenital anomalies of the kidney and urinary tract; CKD, chronic kidney disease; IQR, interquartile range; PTDM, posttransplantation diabetes mellitus.

**FIGURE 2.**
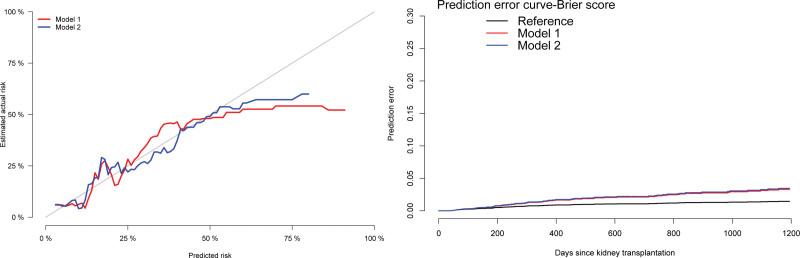
Calibration plot and Brier score of models 1 and 2 (derivation cohort). Calibration and prediction error curve plots for models 1 (red) and model 2 (blue). Calibration curve: y-axis contains the observed risk and x-axis the risk predicted by the model; both are expressed as a percentage. Prediction error curve: y-axis contains the prediction error, and x-axis refers to the d after renal transplantation. An optimal value of <0.25. Model 2 was selected to estimate the risk score.

**FIGURE 3.**
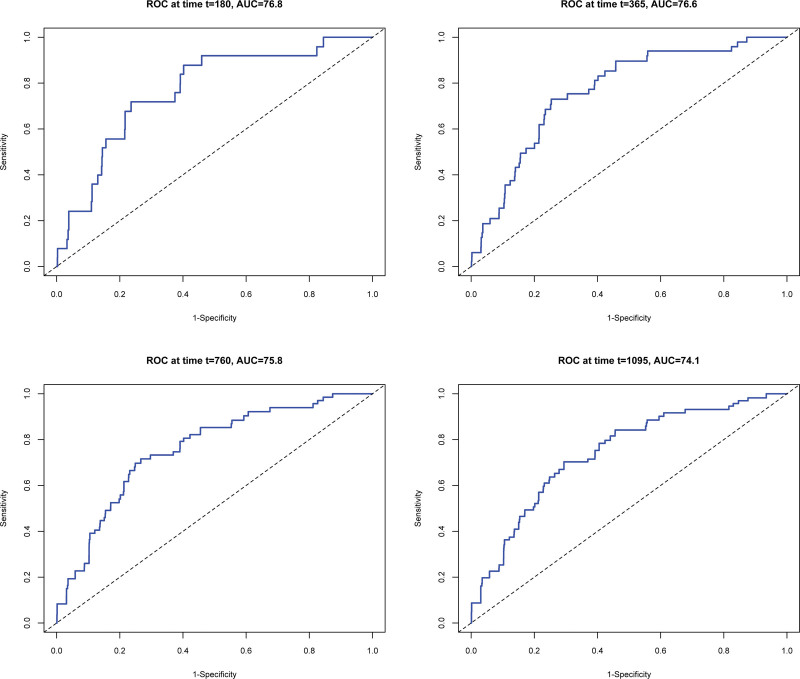
Plot of time-dependent area under the receiver operating characteristics of the score (derivation cohort). Y-axis sensitivity and x-axis 1 – specificity of the risk score model assessed by time-dependent ROC analysis at 6 mo and 1, 2, and 3 y after kidney transplantation. Additionally, the AUC for each specific time was provided with a 95%. AUC, area under the curve; CI, confidence interval; ROC, receiver operator characteristic.

Univariate analysis showed that age and BMI at the time of kidney transplantation, transient posttransplantation hyperglycemia, and the use of tacrolimus were independent predictors of PTDM, whereas cyclosporine was associated with a lower risk of PTDM (Table 2). Based on the univariate analysis and previous testing of collinearity between the independent variables, 4 models were constructed (**Figure S2, SDC,** http://links.lww.com/TXD/A592). The 4 predictive models were compared using the partial likelihood test and the Akaike information criterion criteria, Harrell’s C-index, and CPE, which performed very similarly in all models (**Table S1, SDC,** http://links.lww.com/TXD/A592); the Brier score indicated a prediction error close to zero (optimal value <0.25), and the calibration curve showed a good performance for both model 1 and model 2 (Figure 2). Additionally, model 2 showed better discrimination, with tROC-AUC at 1 and 2 y of 0.77 and 0.76, respectively. Based on this information, a model 2 was selected (Figure 3).

**TABLE 2.** Univariate analysis: outcome PTDM

Predictor	β	SE	_sd_HR	95% CI	*P*
Age	0.046	0.0077	1.047	1.032-1.063	<0.001
Weight	0.017	0.0061	1.017	1.005-1.030	0.004
Body mass index	0.099	0.020	1.104	1.060-1.150	<0.001
Female gender	0.293	0.181	1.341	0.939-1.341	0.106
Fasting blood glucose level^*a*^	0.006	0.001	1.0065	1.004-1.458	<0.001
Hyperglycemia^*a*^	0.609	0.202	1.839	1.238-2.734	0.003
Tacrolimus	0.551	0.189	1.736	1.198-2.515	0.003
Cyclosporine	–0.671	0.213	0.511	0.336-0.776	0.002
Mycophenolate mofetil	0.256	0.198	1.292	0.875-1.909	0.196
Azathioprine	0.843	0.370	0.430	0.888-0.208	0.022
mTOR inhibitor	0.145	0.274	1.156	0.674-1.981	0.596
Induction therapy	–0.158	0.369	0.853	0.414-1.758	0.667

^*a*^Transient posttransplantation hyperglycemia.

β, beta coefficient; CI, confidence interval; mTOR, mammalian target of rapamycin; PTDM, posttransplantation diabetes mellitus; SE, standard error; _sd_HR, subdistribution hazard ratio; SE, standard error.

**FIGURE 4.**
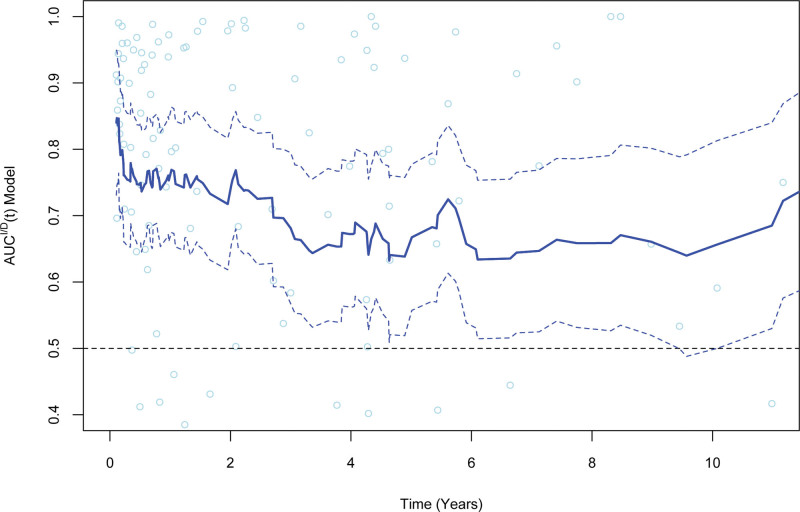
Plot of variation of the AUC during follow-up (derivation cohort). The y-axis shows the AUC value, and the x-axis shows the y of follow-up. The thick blue solid line corresponds to the AUC and the dotted lines correspond to the 95% CI. Circles correspond to PTDM events. The dashed horizontal line corresponds to an AUC value of 0.5 (which means that the probability of the event is equal to the flip of a coin). AUC, area under the curve; CI, confidence interval; PTDM, posttransplantation diabetes mellitus.

Table 3 displays the coefficients, _sd_HR, 95% CI, and *P* values of model 2. This model included 5 variables: age, gender, BMI, transient posttransplantation hyperglycemia, and tacrolimus therapy. This model showed a good discrimination ability with a Harrell’s C-index of 0.70 (95% CI, 0.65-0.76) and a CPE of 0.687 (standard error 0.016). Furthermore, the discriminative ability was similar over time, as shown by the AUC of the time-dependent area under the receiver operating (Figure 3) and by AUC^I/D^ during all follow-ups (Figure 4). The maximum sensitivity and specificity of the model for predicting the outcome of PTDM was achieved during the first y of follow-up. Additionally, the proportionality assumption was tested for all predictors and the *P* value of the overall Schoenfeld residual was 0.1223.

**TABLE 3.** Multivariable time-dependent Cox regression model performed in the derivation cohort: outcome

Model 2/predictor	β	SE	_sd_HR	95% CI	*P*
Age	0.04	0.01	1.04	1.03-1.06	<0.001
Body mass index	0.07	0.02	1.07	1.03-1.12	0.002
Gender, female	0.50	0.19	1.66	1.15-2.38	0.007
Hyperglycemia^*a*^	0.43	0.21	1.54	1.01-2.34	0.046
Tacrolimus	0.61	0.19	1.83	1.26-2.67	0.002

Likelihood-ratio test: 73.42, log partial likelihood: –665.07, AIC: 1332.13.

^*a*^Transient posttransplantation hyperglycemia.

AIC, Akaike information criterion; β, beta coefficient; CI, confidence interval; PTDM, posttransplantation diabetes mellitus; _sd_HR, subdistribution hazard ratio; SE, standard error.

The risk model was calculated using the following formula:

*Score = (0.4 × age) + (0.7 × BMI) + (5 × female gender) + (4 × transient hyperglycemia) + (6 × tacrolimus*)

**FIGURE 5.**
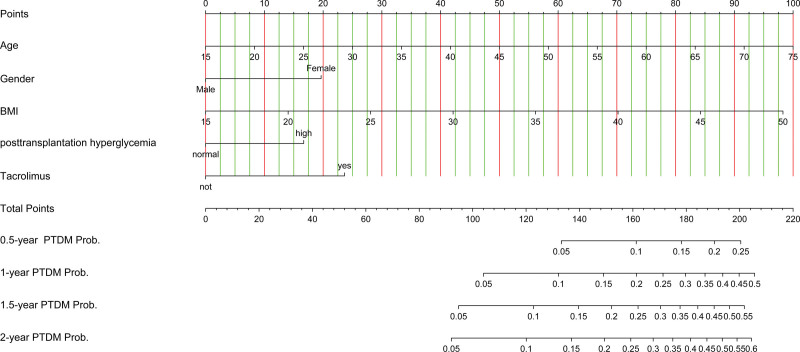
Nomogram for predicting PTDM in patients after kidney transplantation. The nomogram was composed of 4 variables: age (y), gender, body mass index, transient posttransplantation hyperglycemia, and tacrolimus therapy. The nomogram is used as follows: the first horizontal line displays the points; the reader locates how many points each predictor has according to the patient’s characteristics, and then adds up the points and locates the total number of points on the last horizontal line. Finally, the reader draws a vertical line from the total number of points and obtains the probability of the PTDM. For example, a 50-y-old woman (age 58 points; gender 19 points), with a body mass index of 28 (38 points), who presented with hyperglycemia in the first wk after transplantation (17 points) and who received tacrolimus (24 points) = 156. The probability of PTDM is 9% at 6 mo, 18% at 1 y, 22% at 18 mo, and 25% at 2 y. BMI, body mass index; PTDM, posttransplantation diabetes mellitus.

By applying the score in the proportional Cox regression model, the risk of PTDM increased with increasing scores (_sd_HR de 1.09; 95% CI, 1.07-1.012; *P* < 0.0001). Internal validation using the bootstrap method (1000 bootstrap resampling) was used to evaluate the accuracy of model 2. Calibration was obtained in the form of the shrinkage coefficient to quantify the lack of fit of the model. The shrinkage coefficient was 99.7%, indicating a –0.98% lack of fit in the model, whereas Harrell’s C-index corrected was 0.7348, indicating an adequate correlation between the log hazard and the observed survival time. The detailed results of the model validation indices are provided in **Table S2 (SDC,** http://links.lww.com/TXD/A592). We then calculated the 1-, 2-, and 5-y nomogram predictions. The total score of the patients was obtained by summing the scores corresponding to each subvariable to obtain the predicted probability of PTDM at 1, 2, 3, and 5 y after transplantation (Figure 5).

### External Validation

The performance of the proposed model was tested using a cohort of 251 adult renal transplant patients from Tübingen, Germany, of whom 19 were excluded for failure to measure the PTDM result and 34 for lack of measurement of the predictor transient posttransplantation hyperglycemia. A total of 198 patients with complete cases were included in the analysis, of whom 33 had PTDM during follow-up (16.6%). The baseline characteristics of the patients are shown in Table 4. The median age of all patients was 49.9 (42.3–59.6) y, and 82 (41.4%) were female individuals; furthermore, most patients received tacrolimus at the time of renal transplantation, and all cases of PTDM were diagnosed via HbA1c criteria. Our risk prediction model was applied to each patient for external validation. In this cohort, the risk of PTDM increased with increasing score [_sd_HR 1.12 (95% CI, 1.58-1.185, *P* < 0.0001)]. The tROC-AUC at 6, 12, 18, and 24 mo were 0.71, 0.72, 0.73, and 0.73, respectively. The calibration curve showed a good fit (**Figure S3, SDC,** http://links.lww.com/TXD/A592). The sensitivity and specificity of the predictive model at different time points are presented in Table 5. Next, the risk score of the derivation cohort was divided into 4 quartiles. Kaplan-Meier of the scores stratified by quartiles was performed. Participants in the highest quartile had a higher risk of PTDM than those in the lowest quartile (*P* < 0.001; data not shown).

**TABLE 4.** Baseline characteristics, external validation cohort (n = 198)

	Total cohort from Tübingen, Germany		PTDM			
	(N = 198)		Non-PTDM (n = 165)		PTDM (n = 33)	
Variables	Median	IQR	Median	IQR	Median	IQR
Age, y	49.9	42.3–59.6	48.3	40.3–57.1	55.7	47.0–66.8
Weight, kg	72	60.5–81.0	71.5	60.2–80.8	73.0	61.5–82.0
Body mass index, kg/m^2^	24	21.6–26.2	23.9	21.6–26.0	24.9	22.2–26.9
	**n**	**%**	**n**	**%**	**n**	**%**
Gender						
Female	82	41.4	72	43.6	10	30.3
Male	116	58.5	93	56.3	23	69.7
	**n**	**%**	**n**	**%**	**n**	**%**
Hyperglycemia^*a*^	37	18.7	23	13.9	14	42.2
	**n**	**%**	**n**	**%**	**n**	**%**
Tacrolimus	188	94.9	158	95.8	30	90.9

^*a*^Transient posttransplantation hyperglycemia.

IQR, interquartile range; PTDM, posttransplantation diabetes mellitus.

**TABLE 5.** Comparison of test performance characteristics to predict PTDM over time using the median score in derivation and validation cohorts

Performance characteristics	Derivation cohort (Medellín, Colombia) Total number of kidney transplant recipients = 727 Total number of PTDM events = 122					External validation cohort (Tübingen, Germany) Total number of kidney transplant recipients = 198 Total number of PTDM events = 33				
Months^*a*^	6	12	24	36	48	6	12	24	36	48
Observed events^*b*^	25	48	63	69	75	15	26	28	30	30
Se	91.90 (5.49)	89.55 (4.43)	85.36 (4.52)	85.38 (4.35)	81.27 (5.40)	86.79 (8.74)	88.87 (6.09)	89.90 (5.57)	91.05 (4.98)	91.05 (4.98)
Sp	48.54 (2.07)	51.0 (2.24)	53.94 (2.75)	54.09 (3.36)	51.37 (4.14)	37.65 (3.73)	43.18 (4.32)	45.56 (5.26)	50.88 (6.64)	50.88 (7.63)
PPV (SE)	6.69 (1.35)	13.50 (1.93)	18.48 (2.36)	21.41 (2.71)	23.65 (3.08)	10.58 (2.78)	20.68 (3.89)	23.48 (4.3)	28.49 (5.23)	29.62 (5.69)
NPV (SE)	99.33 (0.47)	98.28 (0.76)	96.79 (1.06)	96.19 (1.21)	93.67 (2.05)	97.1 (2.02)	95.9 (2.33)	96.04 (2.27)	96.36 (2.10)	96.53 (2.02)

^*a*^Months posttransplantation.

^*b*^Observed PTDM events at that time.

NPV, negative predictive value; PPV, positive predictive value; PTDM, posttransplantation diabetes mellitus; SE, standard error; Se, sensitivity; SP, specificity.

## DISCUSSION

In this retrospective real-world study, a predictive model of PTDM risk was developed using a survival analysis methodology with time-dependent variables and competing risk analysis. The model included the following predictors: age, gender, BMI, transient posttransplantation hyperglycemia, and tacrolimus therapy. This model showed good predictive performance. The tROC curve showed a stable AUC during the follow-up and indicated that the best discrimination ability occurred during the first y posttransplantation. Finally, external validation in a cohort from a different country with different ethnicities was performed adequately, indicating that this risk prediction model is a sensitive and specific prognostic indicator of PTDM, transferable to other regions.

A predictive model for application in the pretransplant period was published in 2011. The incidence of PTDM was 26.7% (85/318) at the 1-y follow-up and included 7 dichotomous variables such as age 50 y or older, BMI ≥30 kg/m^2^, family history of type 2 diabetes mellitus, fasting blood glucose level ≥100 mg/dL pretransplant, triglycerides ≥200 mg/dL, use of gout medication, and pretransplant assignment to corticosteroid maintenance protocol, with good performance with an AUC-ROC of 0.70 in both the derivation and validation set^17,18^; however, this model did not include transient posttransplantation hyperglycemia, which is a newly recognized strong biomarker for PTDM.

Another predictive model for PTDM was developed in 2022 in 495 renal transplant recipients; the incidence of PTDM was 11.1% (55/495) at 6 y of follow-up, which was lower than previously reported. They also found that the cumulative incidence of PTDM increased over time to 15.3% after 3 y, which is comparable with our findings. The prediction model included 6 dichotomous variables: age 45 y or more, body weight >25 kg/m^2^, tacrolimus blood levels >10 ng/mL during the first 3 mo, transient posttransplantation hyperglycemia, delayed graft function, and acute rejection. The performance was very good, with an AUC-ROC of 0.916 (95% CI, 0.862-0.954; *P* < 0.001), but they did not report whether a calibration test had been performed or how to use this model in clinical practice. Furthermore, this model has not been externally validated.^1^

In line with previous publications,^1,19^ our study showed that transient posttransplantation hyperglycemia was a strong predictor of PTDM. Posttransplantation hyperglycemia is a common early adverse event after renal transplantation, affecting up to 91% of kidney transplant recipients within the first 48 postoperative h.^38^ Notably, despite being thought to be transient in most patients, it reveals that in patients at risk for beta-cell failure in stressful situations, such as the early posttransplant period, high doses of glucocorticoids and calcineurin inhibitors are administered, and the surgery and in-hospital stay may be diabetogenic. Notably, by reducing beta-cell stress via reduction in glucotoxicity through the early application of (basal) insulin, the risk of PTDM can be substantially reduced.^39,40^

BMI is a well-established risk factor for PTDM because of the increase in insulin resistance and an alteration in insulin secretion, triggered or not by factors associated with renal transplantation.^41,42^ Similar to previous studies, we found that BMI was an independent risk factor for PTDM.^3,7,43,44^ BMI is a modifiable risk factor that can be addressed by lifestyle intervention, which is powerful in weight reduction and has proven efficacy in improving glucose metabolism,^45^ even after kidney transplantation.^46^ Bariatric surgery before kidney transplantation may also be considered for high-risk patients.^47^ Furthermore, the use of glucagon-like peptide 1 receptor analogs or dual glucagon-like peptide 1/ gastric inhibitory polypeptide analogs for weight reduction may also be an interesting option for future research.

Tacrolimus was also an important predictor of PTDM in the present study, increasing the risk of PTDM by 1.8-fold, whereas cyclosporine did not show a diabetogenic effect, consistent with previously reported data.^41,43^ Tacrolimus is still recommended as the first-line immunosuppressive drug because of its efficacy in preventing rejection and graft loss^48^ because preservation of graft function is one of the major determinants of cardiovascular and overall mortality in kidney transplant recipients.^49^ Current recommendations on PTDM also argue against modification of immunosuppression based on diabetes risk,^20^ although the option of steroid-free maintenance immunosuppression is discussed controversially^50,51^ and may be an option for immunologically low-risk patients.

In summary, our study has several strengths. To our knowledge, this is the first prognostic prediction model for PTDM in renal transplantation using time-dependent survival analysis and competing risks. Therefore, this model can be applied regularly after transplantation, which may improve patient monitoring and provide a better basis for decision-making regarding changes in therapy and close monitoring of glucose levels. Secondly, our model has also been validated in an independent population from another country. Also, our model uses clinical variables that are easy to measure and available during kidney transplant follow-up; therefore, it can be easily implemented in any kidney transplant center. Furthermore, a user-friendly nomogram that facilitates its daily application was provided. Thirdly, this model includes the effect of immunosuppressive therapy and its changes over time on the risk of PTDM. Renal transplant recipients may receive different immunosuppressive therapies or undergo change in immunosuppressive treatment during follow-up, which could influence the risk of PTDM. Therefore, we used adequate statistical methods to avoid bias resulting from the difference in the duration of follow-up and time of treatment exposure between patients.

Our study also has some limitations. Firstly, because of the retrospective data collection, we could not completely rule out patients with subclinical pretransplant diabetes mellitus. Additionally, no information was collected on a family history of type 2 diabetes mellitus. Secondly, the potential of misclassification bias, given that some patients were diagnosed with glycosylated hemoglobin and others with fasting blood glucose levels, could underestimate or overestimate the incidence of the outcome.^43^ However, in our study, the incidence of PTDM in the derivation and external validation cohorts was comparable, 16.78% versus 16.66%, respectively. Furthermore, although we aimed to evaluate all immunosuppressive therapies used, there were insufficient data to associate mammalian target of rapamycin (mTOR) inhibitors with the outcome; however, the combination of tacrolimus and mTOR inhibitors with the development of PTDM remains controversial in the literature and could be an issue for future research.^41^

## CONCLUSION AND POLICY IMPLICATIONS

This study has 2 important implications for clinical practice. First, this predictive model, shown as a nomogram, is a simple tool that can be implemented by kidney transplant teams to estimate the probability of developing PTDM and can be applied not only at the time of transplantation but also during follow-up. The model helps identify patients at risk and individualize monitoring and preventive strategies, including lifestyle intervention or adaptation of immunosuppressive therapy in some cases, weighing the risk of PTDM against the risk of renal graft rejection in each patient. It also helps physicians to provide more reliable information when advising on the risk of future outcomes.

By identifying the patients most at risk of developing PTDM, specific, personalized pharmacological and nonpharmacological measures could be adopted to address modifiable risk factors that, in the long term, could positively impact cardiovascular risk and renal graft survival in these patients.

## FUTURE RESEARCH ISSUES

The future research issues are the predictive role of tacrolimus and mTOR inhibitor combination therapy in the development of PTDM and lipid profile components as predictors of PTDM and prospective exploration of ≥1 accurate definitions of transient posttransplantation hyperglycemia in the prediction of PTDM.

## ACKNOWLEDGMENTS

The authors thank the Hospital Pablo Tobón Uribe for allowing us to conduct this study and for their help in extracting >100 000 laboratory test data points from all available sources and reliable access to information from medical records.

## Supplementary Material


