# Supplementary material for: Development and Validation of a New Score to Assess the Risk of Posttransplantation Diabetes Mellitus in Kidney Transplant Recipients
Source: Transplant Direct. 2023 Nov 8;9(12):e1558. doi: 10.1097/TXD.0000000000001558 (PMC10635612; doi:10.1097/TXD.0000000000001558)
Supplement: Supplementary file 1 [file txd-9-e1558-s001.pdf]

Supplemental digital content "Results"

Table S1. The 4 candidate multivariable models, outcome Posttransplantation Diabetes Mellitus (PTDM)

	HR	95% CI	*p* value	-2LL	AIC	Harrell C (CI 95%)	Gonen and Herrel (SE)	Schoenfeld
**Model 1**								
Age	1.05	1.03 - 1.06	<0.001	663.01	1328	0.70	0.682 (0.014)	0.13
BMI[a]	1.07	1.02 - 1.12	0.003			(0.64 – 0.76)		
Female gender	1.66	1.16 - 2.39	0.006					
Fasting blood glucose level[b]	1.01	1.00 - 1.01	0.001					
Tacrolimus	1.82	1.25 - 2.64	<0.001					
**Model 2**								
Age	1.04	1.03 - 1.06	<0.001	655.07	1332.1	0.70	0.6866 (0.016)	0.12
BMI[a]	1.07	1.03 - 1.12	0.002			(0.65 – 0.76)		
Female gender	1.66	1.15 - 2.38	0.007					
Hyperglycemia[b]	1.54	1.01 - 2.34	0.046					
Tacrolimus	1.83	1.26 - 2.67	0.002					
**Model 3**								
Age	1.05	1.03 - 1.06	<0.001	664.92	1332	0.75	0.682 (0.015)	0.213
Weight	1.02	1.002 - 1.03	0.021					
Female gender	1.66	1.38 - 3.06	<0.001					
Fating blood glucose level[b]	1.01	1.002 - 1.008	<0.001					
Tacrolimus	1.81	1.24 - 2.63	0.002					
**Model 4**								
Age	1.05	1.03 - 1.06	<0.001	666.99	1336	0.76	0.686 (0.017)	0.213
Weight	1.02	1.003 - 1.03	0.014					
Female gender	2.05	1.38 - 3.06	<0.001					
Hyperglycemia[b]	1.58	1.04 - 2.42	0.034					
Tacrolimus	1.82	1.24 - 2.66	0.002					

[a]BMI, body mass index

[b]Transient posttransplantation hyperglycemia

Fasting blood glucose level (mg/dL) as a continuous predictor and hyperglycemia as a dichotomous predictor**;** **HR**, hazard ratio, -2LL**,** Log partial likelihood; **AIC,** Akaike information criterion; **SE**, standard error; Schoenfeld residual p-value

Table S2. Internal validation bootstrapping (1000 resampling)

	Index original	Training	Test	Optimism	Index corrected	Resample
Shrinkage coefficient	1	1	1.0098	-0.0098	1.0098	1000
Sommer´s D-rank correlation	0.4703	0.4709	0.4703	0.0006	0.4697	1000
Harrell C index	0.7352	0.7355	0.7352	0.0010	0.7348	1000

Figure S1: Sankey plot showing immunosuppressive therapy used during the first 3 years after kidney transplantation

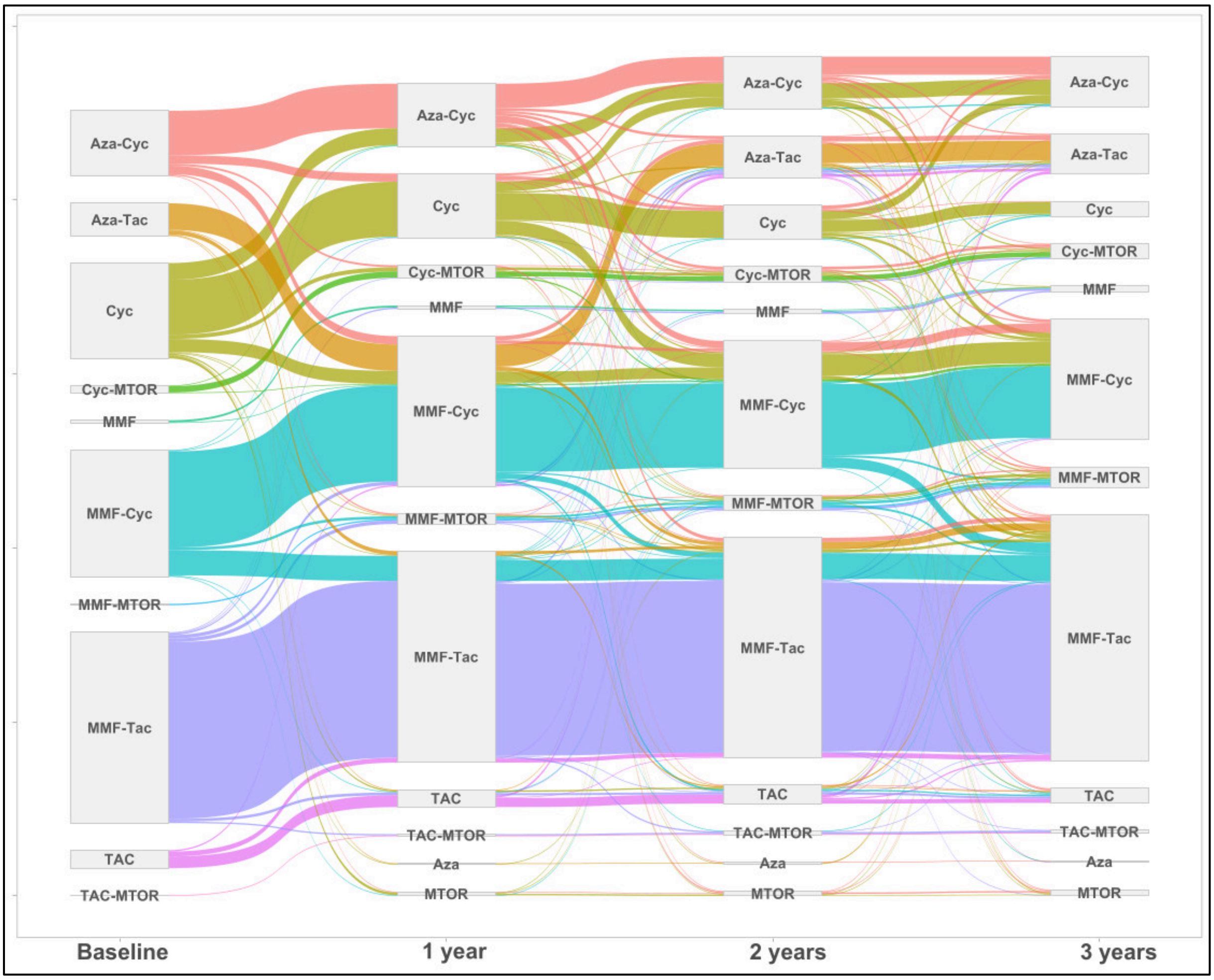

Cyc, Cyclosporine; MMF, Mycophenolate-Mofetil; TAC, Tacrolimus; MTOR-Inhibitor, Sirolimus and Everolimus, Aza; Azathioprine.

Changes in immunosuppressive regimen after renal transplantation are shown. The 4 periods to create the graph were arbitrarily selected and correspond to: baseline between 1 year, first and second year, second and third year of follow-up.

Figure S2: Forest plot of the 4 candidate models developed

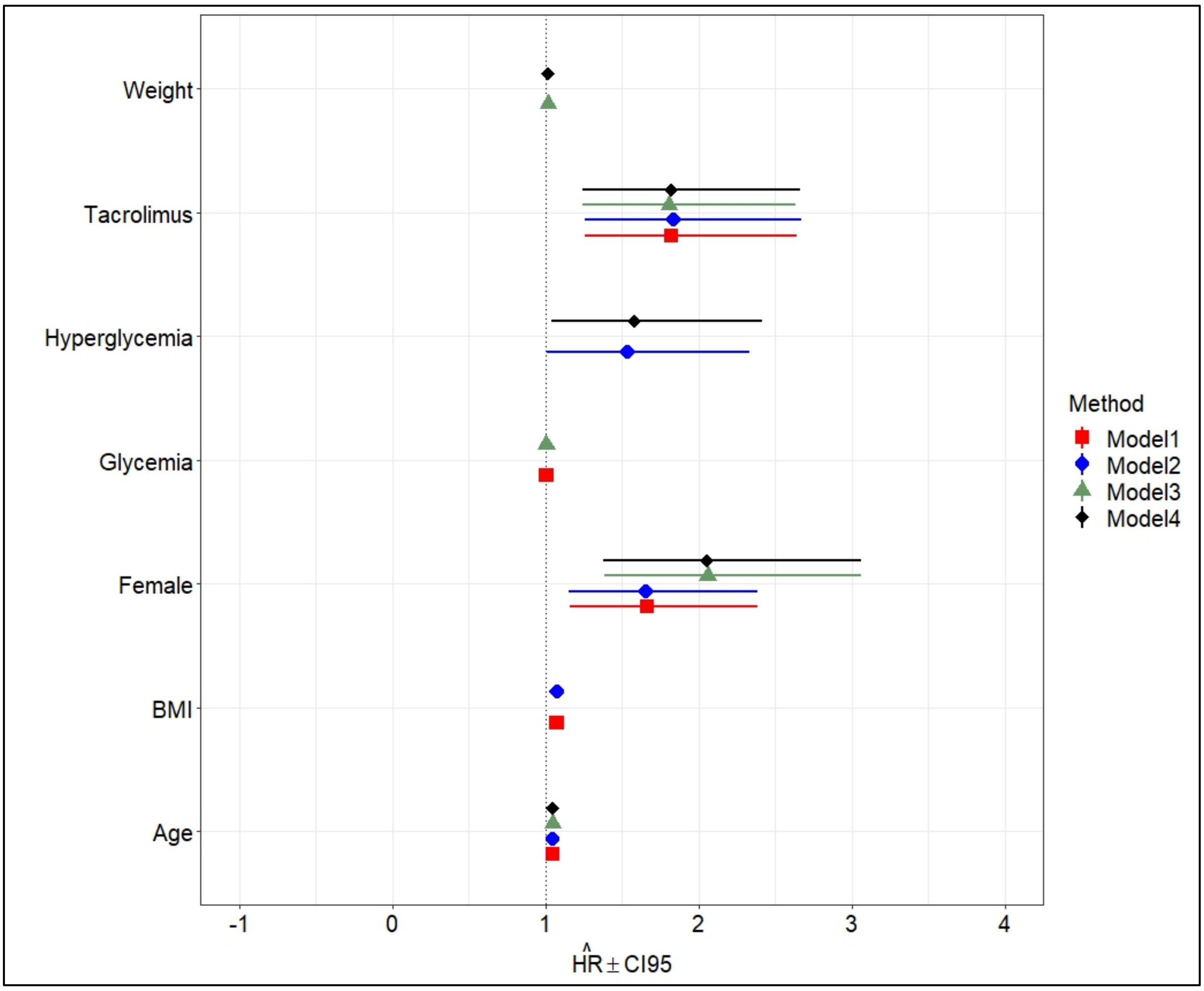

The Y-axis has the predictors (Tacrolimus, weight, Transient posttransplantation hyperglycemia both dichotomous variable (hyperglycemia) and continuous variable (glycemia), female gender, body mass index and age). The X axis has the hazard ratio (HR) and its corresponding 95% confidence interval. The predictor models corresponding to each model are grouped by color.

Selected model for build de score was model number 2

Figure S3. Brier score and Calibration plot (Validation cohort)

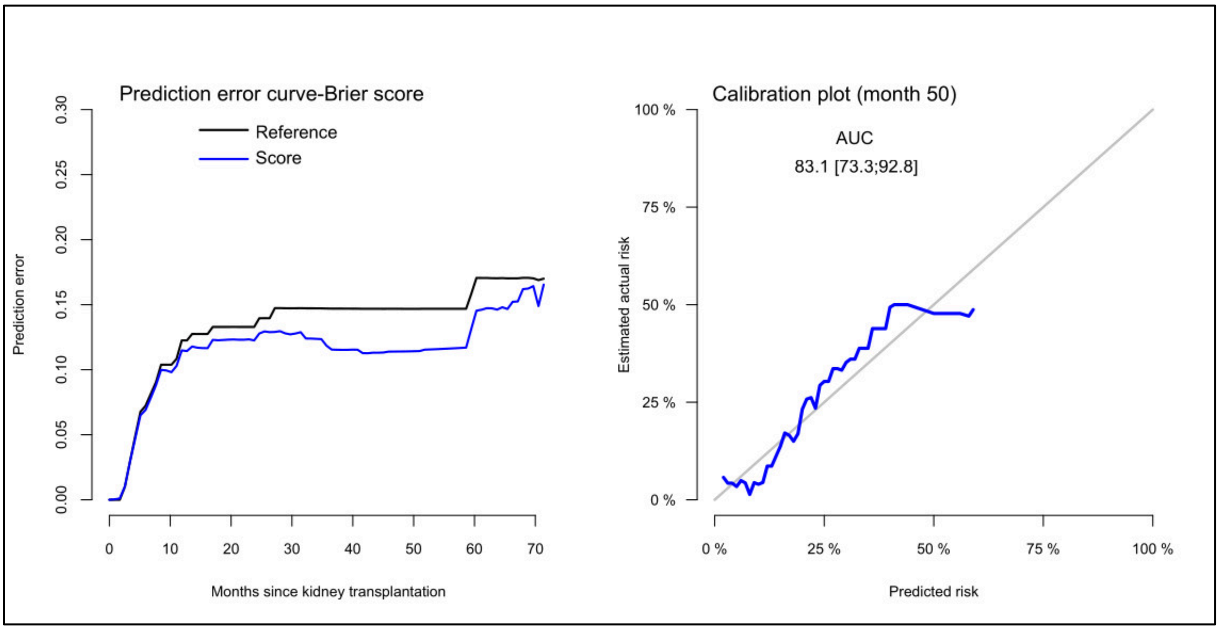

Prediction error curve (in the left side of the figure): Y-axis contains the prediction error and X-axis refers to days after renal transplantation. Optimal value less than 0.25

Calibration graph at 50 months of follow-up (in the right side of the figure): The Y axis contains the observed risk and the X axis the predicted risk by the model; both expressed as a percentage
